# Educating current and future healthcare professionals on evidence-based sustainable medicine

**DOI:** 10.1016/j.clinme.2026.100606

**Published:** 2026-06-11

**Authors:** Katharine Creamer, William K. Gray, Simon Tso, Tim Briggs, Joseph B. John

**Affiliations:** a Getting It Right First Time, NHS England, London, UK; b Warwick Dermatology Centre, South Warwickshire University NHS Foundation Trust, Warwick CV34 4UN, UK

**Keywords:** Education, Sustainable healthcare

## Abstract

Increasing recognition of the need to deliver environmentally sustainable healthcare has led to much new research in this field. Many studies now estimate a ‘carbon footprint’ of an area of healthcare and often go on to highlight potential ways that emissions can be reduced. Before changing their own clinical practice, healthcare professionals must be educated on the methodology and potential for biases of studies in this area. Here, we discuss the general approaches to carbon footprinting, highlight key considerations and suggest future steps which will enable clinicians to deliver evidence-based sustainable medicine.

With increasing recognition of the need to deliver environmentally sustainable healthcare, analyses describing the environmental footprint of healthcare activities will inform decisions in clinical practice. It is essential that healthcare professionals are equipped with an understanding of the methodology, utility and potential biases of studies in this field – enabling them to implement evidence-based sustainable medicine in their own practices and reduce healthcare’s overall contribution to climate change.

A ‘carbon footprint’ gives an estimate of the greenhouse gas (‘carbon’) emissions associated with a product, procedure or pathway. Carbon footprinting studies also often highlight potential ways to reduce carbon emissions. Models based on economic activity (‘environmentally extended input-output models’) convert costs into estimated emissions using carbon emissions characterisation factors for different activities. This ‘top-down’ approach provides rough estimates, but is inaccurate and can distort and over/underestimate results.^1^ A more accurate modelling approach is to undertake process-based cradle-to-grave emissions modelling. This ‘bottom-up’ approach involves measuring all relevant material and energy inputs in a product or service’s life cycle and estimating associated emissions. A ‘hybrid’ approach, involving process-based modelling with cost-based emissions factor substitution to fill data, gaps is sometimes used.

In all models, ‘assumptions’ are used to describe activities and estimate emissions, and boundaries are set stating what will be included in or excluded from the model. Therefore, carbon footprint estimates can vary significantly depending on the methodology used. A study by Kooistra et al.^1^ found a fourfold difference in estimates when using different methods. More importantly, the ‘hotspots’ contributing most significantly to the total emissions, and potentially guiding mitigation actions, varied markedly depending on the modelling approach.

If research suggests that an activity can be safely omitted from clinical care, then modelling its environmental benefit can be straightforward, since carbon emissions estimates can afford to be less accurate. However, where one activity may require substitution for another, then more accurate carbon emissions modelling that compares different options in a like-for-like manner is essential. The generalisability of emissions data to any specific patient population or healthcare setting is a further fundamental consideration.

The GMC^2^ has supported the Education for Sustainable Healthcare (ESH) curriculum^3^ integrating teaching on sustainable healthcare into UK medical education. Previous research^4^ has highlighted strong interest among healthcare professionals in reducing the carbon footprint of the NHS, but identified concerns over limited formal training. Greener NHS has identified environmentally sustainable models of clinical care as a key priority in their roadmap for change.^5^ This may require clinicians to consider changing their clinical practice based on negative environmental impact. We encourage training about critical appraisal of sustainability research to empower future doctors with the tools they require to design and deliver sustainable healthcare.

The practical matter of how to upskill healthcare professionals in understanding sustainability research is likely to require a multifaceted approach. Integrating sustainability into medical school teaching and resident doctor training curricula are necessary. Annual specialty meetings offer opportunities to present research on sustainable healthcare, and some conferences have incorporated ‘green prizes, to recognise projects with environmental benefit. Funding opportunities for research into climate, health and environmental sustainability are now available from bodies such as the National Institute for Health and Care Research, UK Research and Innovation and the Wellcome Trust. These are likely to drive the standard of research and general awareness up.

Ongoing data-informed decarbonisation is essential to achieve a net-zero NHS. There is a clear need to educate current and future health professionals on evidence-based sustainable healthcare to support this aim.

## CRediT authorship contribution statement

**Joseph B. John:** Writing – review & editing, Supervision. **Tim Briggs:** Supervision. **Simon Tso:** Writing – review & editing, Supervision. **William K. Gray:** Writing – review & editing, Supervision. **Katharine Creamer:** Writing – review & editing, Writing – original draft, Conceptualization.

## Funding

This research did not receive any specific grant from funding agencies in the public, commercial, or not-for-profit sectors.

## Declaration of Competing Interest

The authors declare that they have no known competing financial interests or personal relationships that could have appeared to influence the work reported in this paper.
